# Response to Letter Regarding “Multidisciplinary Timely Debrief Sessions after Cardiac Arrests and Endotracheal Intubations in the Pediatric Emergency Department”

**DOI:** 10.1097/pq9.0000000000000917

**Published:** 2026-09-28

**Authors:** Jonathan A. Hermel, Yvette Wang, Jamie M. Klapp, Amy Bryl, Martha Pannepacker, Elise Zimmerman

**Affiliations:** From the * Division of Neonatology, Department of Pediatrics, UCLA David Geffen School of Medicine, Los Angeles, Calif.; † Department of Pediatrics, University of California, San Diego, Calif.; ‡ Department of Pediatrics, Rady Children’s Hospital San Diego, San Diego, Calif.; § Department of Emergency Medicine, Rady Children’s Hospital San Diego, San Diego, Calif.

We thank the author for the thoughtful comments regarding our quality improvement initiative, “Multidisciplinary timely debrief sessions after cardiac arrests and endotracheal intubations in the pediatric emergency department,”^1^ and appreciate the opportunity to discuss our work further. We are grateful that the author recognizes both the importance of multidisciplinary debriefing and the practical value of implementing a structured process within a high-acuity pediatric emergency department.

We agree that important questions remain regarding the impact of structured debriefing on patient outcomes, team performance, provider well-being, and long-term sustainability. Our initiative, however, was intentionally designed as an implementation-focused quality improvement project. Before evaluating downstream clinical, educational, or provider-centered outcomes, we first sought to establish whether structured multidisciplinary debriefing could be successfully and sustainably integrated into routine clinical practice without disrupting clinical workflow or patient care. Demonstrating successful implementation and sustained adoption was therefore our primary objective rather than establishing the clinical effectiveness of debriefing itself.

We also agree that assessing debrief quality and fidelity using validated assessment instruments would strengthen future investigations. Our intervention was developed using established debriefing methodologies, although formal evaluation of implementation fidelity in our setting was beyond the scope of this initiative. Our REDCap debrief form incorporated the established plus-delta framework, originally described in the DISCERN hot debriefing literature,^2^ and included an embedded facilitation script adapted from the widely recognized “debriefing with good judgment” framework.^3^ These elements provided a standardized structure while promoting psychologically safe reflection and supporting facilitators with varying experience. We agree that effective debriefing extends beyond an unstructured discussion, and our intervention was designed to facilitate consistent, structured conversations while remaining practical for routine clinical care.

Importantly, our intervention was designed to balance standardization with flexibility. We provided a structured framework with scripted prompts and a recommended duration of 7 minutes or less to promote consistent debriefing while allowing teams to adapt discussions to the clinical context and participants’ emotional needs. This approach supported clinicians with limited formal training in debrief facilitation without creating an overly rigid process that might increase implementation burden, discourage participation, or diminish the peer support and collegial reflection that often follow emotionally significant clinical events. Because our primary objective was to establish routine adoption of debriefing in a setting where structured debriefs had not previously been part of standard practice, we prioritized feasibility and psychological safety.

Ultimately, we view our findings as an essential first step toward embedding structured postevent reflection into routine pediatric emergency care. Establishing a sustainable culture in which multidisciplinary teams consistently pause to reflect after critical events is, in our view, a prerequisite to rigorously evaluating which debriefing characteristics most effectively improve patient care, team learning, and provider well-being in our setting. We appreciate the author’s thoughtful observations and agree that future investigations should build upon successful implementation by evaluating patient outcomes, debrief quality, provider well-being, and long-term sustainability.
